# Incidental Solitary Myofibroma in a Young Adult Female Patient: A Case Report and Comprehensive Literature Review of Myofibroma in Adults

**DOI:** 10.7759/cureus.72229

**Published:** 2024-10-23

**Authors:** Anas As'ad, Samer A Al-Shbailat, Hamza Alhawamleh, Husam A Alsalamat

**Affiliations:** 1 General Surgery, Faculty of Medicine, Al-Balqa' Applied University, Al-Salt, JOR; 2 Surgery, Tafila Governmental Hospital, Al-Tafila, JOR; 3 Surgery, Faculty of Medicine, Jordan University of Science and Technology, Irbid, JOR; 4 Biopharmaceutics and Clinical Pharmacy, University of Jordan, Amman, JOR

**Keywords:** incidental finding, jordan, myofibroma, neck mass, surgery

## Abstract

Myofibroma is a rare mesenchymal tumor typically observed in children, with only a few reported cases in adults. It can be easily mistaken for more common benign lesions, making it essential to include them in the differential diagnosis of soft tissue masses. This case report presents a rare instance of myofibroma in a young adult, and a comprehensive review of the literature presenting case reports and case series of myofibroma cases in the head and neck regions of adult patients aged > 18 years.

A 22-year-old woman presented with a gradually enlarging, painless mass on the right side of her neck which had been developing over the course of two years. An ultrasound initially identified a 1×2 cm lipoma; however, during surgery, a fibrous soft tissue mass was discovered extending beneath the sternocleidomastoid muscle. The mass was found to be closely associated with the posterior auricular nerve. Despite its proximity to the nerve, the tumor was successfully excised, while preserving the nerve. Histopathological analysis confirmed a diagnosis of myofibroma. This case highlights the rare occurrence of myofibromas in adults and emphasizes the importance of considering this diagnosis when evaluating soft-tissue masses. Even when imaging suggests a benign lesion, surgical intervention may yield unexpected results. Accurate diagnosis through histopathology is essential, and careful surgical techniques, including nerve preservation, play a critical role in successful management. Long-term follow-up is necessary to ensure the absence of tumor recurrence.

## Introduction

Myofibroma is a rare tumor that remains the most common fibrous tumor in childhood [1]. Adult individuals have also been reported to develop myofibromas; however, the frequency of myofibromas in this population is not well understood. Myofibromas (solitary) and myofibromatosis (multicentric) are benign soft tissue tumors characterized by perivascular myoid differentiation. They usually present as firm, flesh-colored to purple solitary or multiple myofibromas, and are usually painless unless there is nerve compression [2, 3]. Recognizing the occurrence of these tumors in both children and adults, the World Health Organization (WHO) incorporated the terms "myofibroma" and "myofibromatosis" into its 2002 classification of soft tissue tumors [2]. Since then, additional cases involving adults have been reported, with most tumors found in the dermis, subcutaneous tissue, and oral cavity and less frequently in deeper tissue locations.

Despite its rare occurrence, it is important to consider myofibromas in the differential diagnosis of soft tissue masses, even in older patients. In this paper, we describe a rare incidental case of solitary myofibroma in an adult, with the aim of raising awareness of this uncommon entity and highlighting the importance of accurate diagnosis and management.

## Case presentation

Demographic information

A 22-year-old female patient presented with a nontender mass on the right side of her neck. The patient reported a history of gradual enlargement of the mass over the past two years, with a noticeable increase in size in recent months. She had no significant past medical history and denied any other associated symptoms, such as pain, fever, weight loss, or night sweats.

Clinical findings

On April 27, 2023, the patient underwent a comprehensive clinical evaluation. A firm, mobile, non-tender mass measuring approximately 1×2 cm was palpable on the right side of the neck. There was no evidence of overlying skin changes, signs of inflammation, or regional lymphadenopathy. The remaining physical examination results were unremarkable, and no additional abnormalities were detected.

Diagnostic assessments

Considering the patient's age, duration, and mass characteristics, ultrasonography (U/S) was performed on April 27, 2023. Ultrasound imaging revealed a well-circumscribed, 1×2 cm soft tissue mass, which appeared hypoechoic with a homogenous texture, suggesting a lipoma. Despite the benign features seen on ultrasonography, the progressive enlargement of the mass prompted further evaluation and management.

Therapeutic interventions

Owing to the persistent and enlarging nature of the mass, surgical excision was deemed necessary for diagnostic clarification and therapeutic relief. The procedure was conducted on April 29, 2023, under local anesthesia and sedation. Intraoperatively, an unexpected finding of a fibrous soft-tissue mass extending deep beneath the right sternocleidomastoid muscle was noted. The mass was also intimately associated with the posterior auricular nerve, which complicated the excision process. Careful and meticulous dissection was performed to excise the mass while preserving nerve integrity. The excised specimens were subjected to histopathological examination.

Follow-up and outcomes

**Figure 1:**
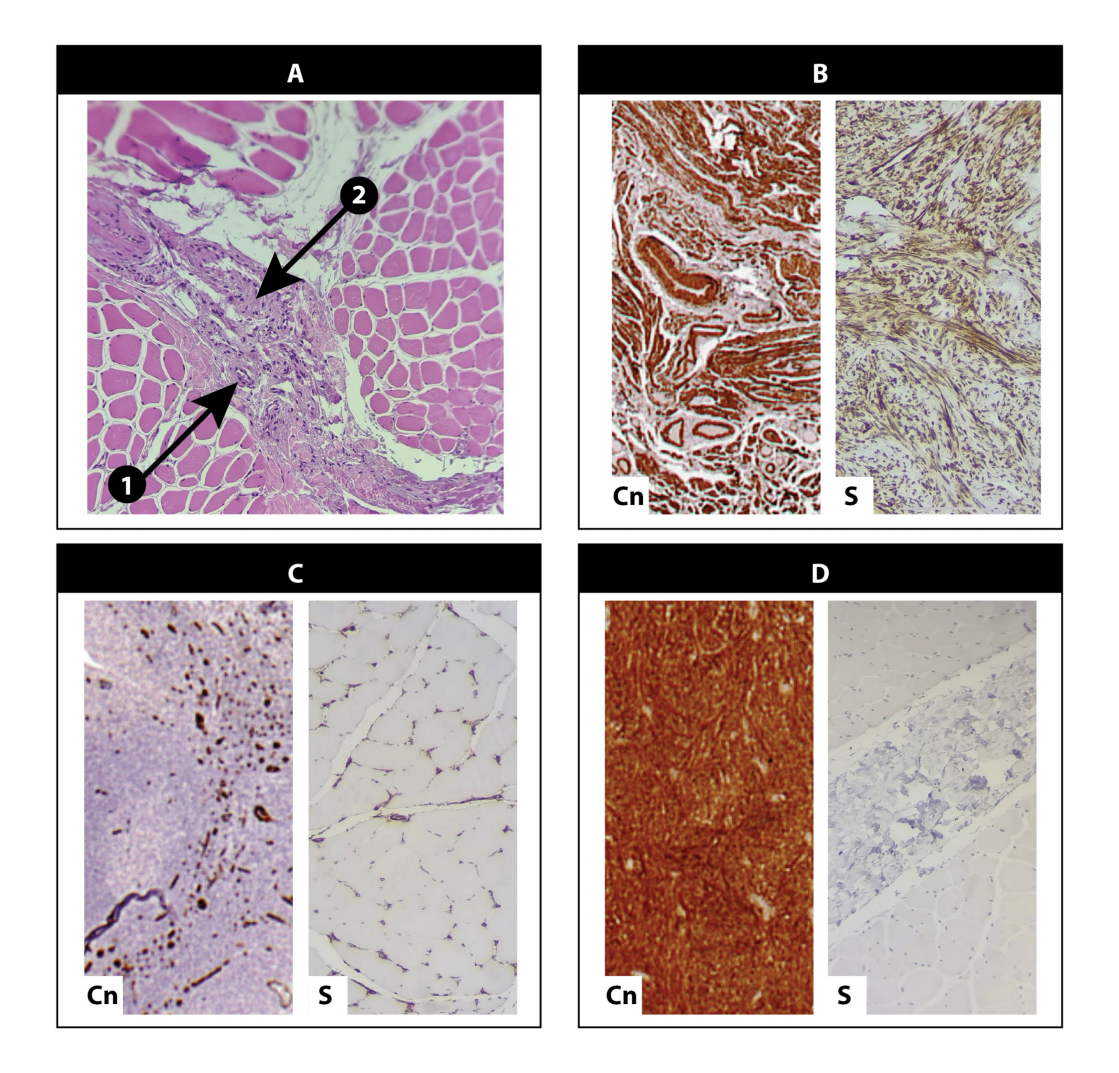
Histopathology image of excised biopsy showing myofibroma features with appropriate immunohistochemistry stains. A: Hematoxylin and eosin stain showing spindle cells arranged in fascicles, the cells are embedded in a collagen-rich matrix; Arrow 1: Collagen matrix; Arrow 2: Spindle cells arranged in fascicles; B: Smooth muscle actin stain, showing smooth muscle actin positivity; C: CD34 stain, negative in comparison to the control; D: S100 stain, negative in contrast to the control. Abbreviations: S, sample; Cn, control

The histopathological examination, completed on May 3, 2023, revealed the proliferation of spindle-shaped cells with abundant eosinophilic cytoplasm arranged in fascicles and nodules. These histological features are indicative of myofibromatosis, a rare low-grade mesenchymal neoplasm. Figure 1 represent images from histopathology of the excised biopsies with the appropriate immunohistochemistry stains.

**Figure 2:**
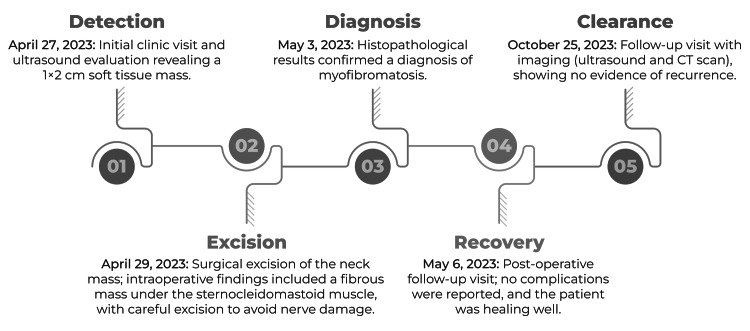
Timeline of the study from detection to clearance

The postoperative follow-up was conducted on May 6, 2023. The patient reported no postsurgical complications and the surgical wound healed appropriately. Follow-up imaging was scheduled to monitor for recurrence. A neck ultrasound was performed six months postoperatively, and a CT scan of the neck was performed one year after surgery on October 25, 2023. Neither imaging modality showed evidence of recurrence, indicating a successful surgical outcome, with no immediate signs of myofibromatosis recurrence. Figure 2 presents a summary of the case timeline, from detection to clearance.

## Discussion

Myofibroma is a rare mesenchymal tumor arising from abnormal myofibroblastic proliferation. It typically follows a benign course and shares several morphological features with myopericytomas, both of which are classified under the same group according to the WHO classification of soft tissue tumors [2]. This tumor predominantly affects pediatric patients, with the head and neck regions being the most commonly affected, particularly the oral region. Both familial and sporadic forms have been previously described. The familial form of myofibromatosis follows an autosomal dominant inheritance pattern and typically involves multiple myofibromas. By contrast, solitary myofibromas are usually sporadic.

After reviewing the English literature, we found numerous case reports and series describing myofibromas in the head and neck regions of adults aged > 18 years. Most adult cases involve the head and neck, although other body regions are also occasionally affected. We reviewed 30 articles that revealed that most cases were solitary. The tumor has been reported in various locations, including the salivary glands, mandible, gingiva, buccal mucosa, tongue, oropharynx, lips, sublingual region, cheeks, eyelids, orbits, ears, palate, periocular regions, and neck.

Torres et al. [4] described 68 cases of salivary gland myofibromas, 63 of which involved adults aged > 18 years. Of these, 53/68 were located in the parotid gland. Similarly, Foss and Ellis [5] described 79 cases of oral myofibromas, with 42 patients being in their third decade of life or older. However, the exact age of all patients was not specified. Two patients presented with multiple masses, with mass sizes ranging from 0.5 to 5.0 cm.

Jin et al. [6] analyzed 15 adult myofibroma cases diagnosed between 2014 and 2020 at the Department of Pathology, Fudan University Shanghai Cancer Center. The cohort included seven males and eight females, with ages ranging from 22 to 74 years. In this group, only three cases involved the head and neck region, while one was located in the vertebral canal of C6-C7. A total of 14 cases were solitary and one was multifocal. Most patients present with a slow-growing, painless subcutaneous nodule, typically 1-2 cm in size.

Pereira de Oliveira et al. [7] reviewed 22 cases of myofibroma (14 men and 8 women), with ages ranging from 1 to 46 years (mean: 19.8 years). The tumor was most commonly found in the mandible (10/22), gingiva (8/22), alveolar ridge (1/22), tongue (1/22), maxilla (1/22), and the submandibular region (1/22). All the patients underwent complete excision. A summary of the articles reviewed in this study is presented in Table 1.

**Table 1:** Articles from the literature presenting case reports and case series of myofibroma cases in the head and neck regions of adult patients older than 18 years of age. MF: myofibroma, S: solitary, NA: not Available F: female, M: male

Case	Author	Age (years)	Sex	Location	Size (cm)	Type of MF	Treatment
1	Oudijk et al. [8]	46	F	Tongue	2.3	S	Excision not clear
2		70	F	Tongue	1	S	Biopsy
3		24	M	Mandible	3	S	Excision not clear
4		55	M	Tongue	NA	S	Excision
5		24	M	Ear	NA	S	Excision
6		70	M	Cheek	1.5	S	Excision
7		22	F	Lip	0.6	S	NA
8	Montgomery et al. [9]	42	F	Tongue	1	S	NA
9		29	M	Retromolar	1.8	S	NA
10		46	F	Retromolar	2.2	S	NA
11		50	M	Gingiva	2.2	S	NA
12		27	F	Palate	1	S	NA
13	Satomi et al. [10]	18	F	Mandible	2.6×2.5	S	Resection
14	Beham et al. [11]	60	M	Gingiva	0.5	S	Excision
15		64	M	Lower eyelid	1.1	S	Excision
16		41	F	Tongue	2.5	S	Excision
17		59	F	Neck	0.6	S	Excision
18		37	M	Left eyebrow	1	S	Excision
19	Jones et al. [12]	70	F	Gingiva	0.8×0.5	S	NA
20		35	M	Mandible	0.7×0.5	S	NA
21		19	M	Mandible	2.5×1.2	S	NA
22		25	F	Buccal mucosa	0.8×0.5	S	NA
23		46	F	Lower lip	1.0×0.8	S	NA
24		53	M	Buccal mucosa	2.0×2.0	S	NA
25		55	F	Tongue	0.3×0.4	S	NA
26	Jennings et al. [13]	25	M	Sublingual	NA	NA	NA
27	Daimaru et al. [14]	35	M	Buccal mucosa	0.9×2.2	S	Excision
28	Sahin et al. [15]	77	M	Tongue	2.0×1.5	S	Resection
29	Speight et al. [16]	41	F	Tongue	2.5	S	NA
30	Ugar et al. [17]	21	M	Mandible	2.0×1.5	S	Excisional biopsy
31	Oliver et al. [18]	34	F	Mandible	2.5×1.5	S	NA
32	Sedghizadeh et al. [19]	20	M	Mandible	NA	S	Excision
33	Ramadorai et al. [20]	32	F	Mandible	4.2×2.0	S	Excision
34	Lyons et al. [21]	28	M	Mandible	NA	NA	NA
35	Brierley et al. [22]	43	F	Mandible	1	S	Curettage and teeth extraction
36	Lin et al. [23]	44	F	Oropharynx	3.0×1.7×1.4	S	Excision
37	Hemlatha A L et al. [24]	26	F	Left orbit	3.0×1.5	NA	NA
38	Dray et al. [25]	35	F	Left posterior neck	1	S	NA
39	Davies et al. [26]	34	F	C6-7 facet joint	NA	S	Excision
40	Asirvatham et al. [27]	18	M	The axis and odontoid process	NA	S	C2 curettage
41	Swierkowski et al. [28]	48	M	C7-T1 intervertebral foramen	10 mm3	S	Excision
42	Hoang et al. [29]	25	M	Skull base	NA	S	Excision
43	Servat et al. [30]	47	M	Superior portion of the orbit, right frontal sinus and the right frontal lobe	8.0×8.0×2.5	S	Intraorbital and frontal tumor resected
44	Choopong et al. [31]	19	F	Supranasal epibulbar of the left eye	0.6×0.7×0.2	S	Excisional biopsy
45	Heath et al. [32]	71	M	Right lower eyelid	2	S	Excision
46	Morrow et al. [33]	24	F	Left orbit	NA	S	Excisional biopsy

In infantile myofibromatosis (IM), histopathological features include interconnected bundles, nodules, and coiled arrangements of spindle-shaped myoid cells within a matrix of myxoid and collagenous stroma [34]. Immunohistochemical staining is typically positive for smooth muscle actin, vimentin, and occasionally CD34 [34]. At the molecular level, germline or somatic heterozygous mutations in PDGFRB have been identified as the underlying cause of infantile myofibromatosis [35, 36]. PDGFRB, located on the 5q32 locus, encodes platelet-derived growth factor receptor beta (PDGFR-β), a cell surface receptor with tyrosine kinase activity that plays a critical role in embryogenesis and development [37]. A recent large-scale study, which included 69 patients with myofibroma, did not identify PDGFRB mutations in tumors in patients older than 18 years [38].

## Conclusions

This case highlights the importance of considering rare differential diagnoses, such as myofibroma, in young adults presenting with longstanding neck masses. Although initial imaging suggested lipoma, intraoperative findings and histopathology confirmed the diagnosis of myofibroma. Accurate diagnosis is critical for guiding appropriate management and improving patient outcomes. Continued reporting and research on such rare cases will enhance the understanding and clinical approach to myofibromas in similar patient populations.
